# Accumulation of APP-CTF induces mitophagy dysfunction in the iNSCs model of Alzheimer’s disease

**DOI:** 10.1038/s41420-021-00796-3

**Published:** 2022-01-10

**Authors:** Seung-Eun Lee, Daekee Kwon, Nari Shin, Dasom Kong, Nam Gyo Kim, Hee-Yeong Kim, Min-Ji Kim, Soon Won Choi, Kyung-Sun Kang

**Affiliations:** 1 Adult Stem Cell Research Center and Research Institute for Veterinary Science, College of Veterinary Medicine, Seoul National University, Seoul, 08826 Republic of Korea; 2 Research Institute in Maru Therapeutics, Seoul, 05854 Republic of Korea

**Keywords:** Reprogramming, Energy metabolism, Neural stem cells

## Abstract

Mitochondrial dysfunction is associated with familial Alzheimer’s disease (fAD), and the accumulation of damaged mitochondria has been reported as an initial symptom that further contributes to disease progression. In the amyloidogenic pathway, the amyloid precursor protein (APP) is cleaved by β-secretase to generate a C-terminal fragment, which is then cleaved by γ-secretase to produce amyloid-beta (Aβ). The accumulation of Aβ and its detrimental effect on mitochondrial function are well known, yet the amyloid precursor protein-derived C-terminal fragments (APP-CTFs) contributing to this pathology have rarely been reported. We demonstrated the effects of APP-CTFs-related pathology using induced neural stem cells (iNSCs) from AD patient-derived fibroblasts. APP-CTFs accumulation was demonstrated to mainly occur within mitochondrial domains and to be both a cause and a consequence of mitochondrial dysfunction. APP-CTFs accumulation also resulted in mitophagy failure, as validated by increased LC3-II and p62 and inconsistent PTEN-induced kinase 1 (PINK1)/E3 ubiquitin ligase (Parkin) recruitment to mitochondria and failed fusion of mitochondria and lysosomes. The accumulation of APP-CTFs and the causality of impaired mitophagy function were also verified in AD patient-iNSCs. Furthermore, we confirmed this pathological loop in presenilin knockout iNSCs (PSEN KO-iNSCs) because APP-CTFs accumulation is due to γ-secretase blockage and similarly occurs in presenilin-deficient cells. In the present work, we report that the contribution of APP-CTFs accumulation is associated with mitochondrial dysfunction and mitophagy failure in AD patient-iNSCs as well as PSEN KO-iNSCs.

## Introduction

Alzheimer’s disease (AD) is a progressive neurodegenerative pathology defined as the accumulation of hyperphosphorylated tau (p-tau) protein aggregates within neurons and extracellular amyloid-beta (Aβ), a product of amyloid precursor protein (APP) processing [1]. Mutations in the *APP, Presenilin 1 (PSEN1)*, and *Presenilin 2 (PSEN2)* genes are known to be a cause of early-onset forms of Alzheimer’s disease, termed familial Alzheimer’s disease (fAD) [2]. APP can be cleaved into two different pathways, amyloidogenic and nonamyloidogenic. Under normal physiological conditions, APP can be processed by α- and γ-secretases. However, during the amyloidogenic pathway, APP is sequentially cleaved by β- and γ-secretases to produce N-terminal fragment of APP (sAPPβ) and C-terminal fragments (CTFβ, C99), and C99 further processes Aβ38, Aβ40, and Aβ42 fragments, which are the major pathologies of AD [3].

In recent decades, most AD research has focused on Aβ, a final product of APP processing, which has a neurotoxic effect in the brain, and most clinical trials have targeted Aβ. However, only a few studies have shown that CTF accumulation in APP processing is neurotoxic and causes synaptic loss, leading to long-term memory impairment in several AD models [4–6]. Transgenic mice expressing C99 fragments or injected with CTFβ showed impaired synaptic plasticity, long-term memory, and astrogliosis [7, 8]. The accumulation of CTFs is also detected in human cerebrospinal fluid (CSF), and the level of CSF is higher in AD patients who have PSEN1 mutations [9]. It is also suggested that CTFs could be a potential diagnostic biomarker as an early neuropathological feature for AD.

Mitochondria are basic organelles that perform multiple important functions in various cellular processes. Many neural activities consume energy in the brain, and mitochondria are the major energy source that provide ATP through oxidative phosphorylation to maintain normal neuronal function and activity. To date, many studies have been established on impaired energy metabolism in the brains of AD patients. Consistent with the observation that damaged energy metabolism continues prior to the clinical onset of AD, abnormal mitochondria has been established as an early sign of proceeding AD and contributing to the progression of the disease [10]. Most AD-related mitochondrial dysfunction involves impaired oxidative phosphorylation (OXPHOS) activity, decreased ATP levels, and increased ROS levels [11].

Mitochondrial homeostasis is controlled by mitophagy, a selective autophagy mechanism for eliminating damaged or functionally altered mitochondria [12, 13]. One of the major processes of mitophagy is the PTEN-induced kinase 1 (PINK1) and E3 ubiquitin ligase (Parkin)-mediated pathway, where Parkin is recruited to the mitochondria and modulates mitochondria dynamics and their degradation through autophagy [14]. After mitochondrial damage, PINK1 recruits Parkin to produce polyubiquitinated proteins of autophagy adapter, including sequestosome-1 (SQSTM1/p62) [15]. Furthermore, interaction with microtubule-associated protein 1 light chain 3 (LC3) mediates the engulfment of cytosolic cargo such as damaged organelles into autophagosomes for a final recycling step by fusion with lysosomes [16].

Recently, AD-related mitophagy failure has been reported based on the accumulation of autophagic vesicles linked to Aβ and p-Tau [17–20]. Moreover, it has also been demonstrated that intraneuronal APP-CTFs trigger early neurotoxicity, mitochondrial abnormalities, and mitophagy dysfunction in 3xTg-AD mice and sporadic AD patients [21–23]. PSEN is one of the γ-secretase complexes involved in the amyloid cascade [24], and the effect of APP-CTFs accumulation related to PSEN function has not yet been demonstrated.

To investigate whether the effects of APP-CTFs accumulation on mitophagy in Alzheimer’s disease were related to PSEN mutation, we generated induced neural stem cells (iNSCs) from fAD patient-derived fibroblasts, as described in our previous study [25, 26]. Because terminally differentiated neurons derived from the iNSC lineage are very useful for understanding the pathophysiological mechanisms of many neurodegenerative diseases [27], we generated AD patient-iNSCs to study the pathological mechanism of AD. AD patient-iNSCs carrying fAD mutation showed a defect in the degradation step of autophagy process associated with APP-CTFs accumulation, resulting in the accumulation of dysfunctional mitochondria, which was consistent with mitophagy impairment. We also demonstrated mitochondrial dysfunction and impaired autophagy in the absence of PSEN using gene editing in iNSCs to verify the critical role of PSEN in AD. Taken together, this study investigates the mitochondrial function and mitophagy associated with APP-CTFs, which could underlie early-stage features of AD pathology using patient-derived iNSCs and PSEN KO-iNSCs.

## Results

### Altered localization and dysfunction of mitochondria in AD patient-iNSCs

**Fig. 1:**
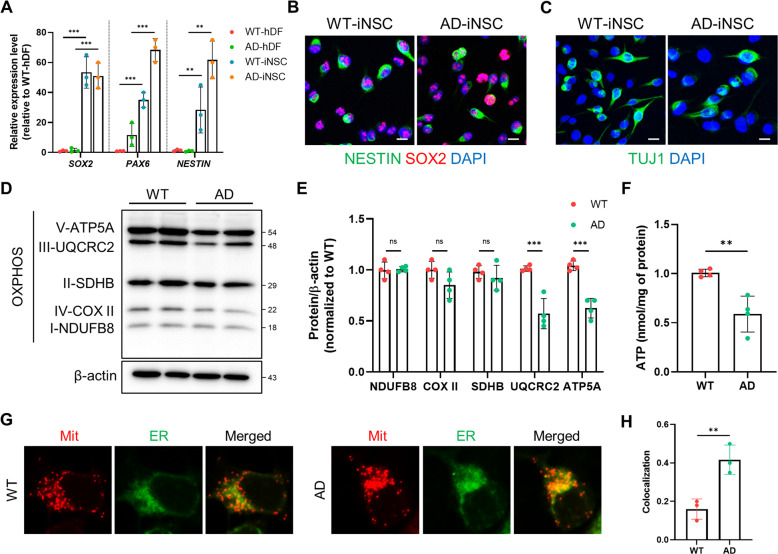
Mitochondria abnormalities in AD-iNSCs. **A** mRNA expression of neural progenitor-related markers using qRT–PCR. The relative expression levels of *SOX2, PAX6*, and *NESTIN* in WT-hDFs, AD-hDFs, WT-iNSCs, and AD-iNSCs. **B** Immunostaining of NESTIN (green) and SOX2 (red) in WT- and AD-iNSCs. Nuclei were detected with DAPI. Scale bar, 10 µm. **C** Immunostaining of TUJ1, an early neuron marker, in WT- and AD-iNSCs. **D** Western blotting of mitochondrial oxidative phosphorylation subunits (OXPHOS) in total cell extracts of WT- and AD-iNSCs using OXPHOS cocktail antibodies. **E** Quantification of the levels of ATP5A, UQCRC2, SDHB, COX-II, and NDUFB8 in WT- and AD-iNSCs. **F** The levels of ATP production in WT- and AD-iNSCs. **G** Mitochondria (Mit) and ER distribution of WT- and AD-iNSCs stained with MitoTracker and ER-Tracker. **H** Quantification of colocalization using ImageJ in WT- and AD-iNSCs. Statistical analysis was performed by Student’s *t*-test. *P < 0.05, **P < 0.01, and ***P < 0.001. The results are presented as the means ± SD.

Mitochondrial abnormalities of OXPHOS of electron transport have been reported to be associated with AD. To investigate whether the mitochondrial function is dysregulated in AD neurons, we established iNSC-derived neuronal cultures using a familial AD cell line. To identify the characteristics of the generated iNSCs, the relative transcription levels of NSC markers including *PAX6, SOX2*, and *NESTIN*, in WT- or AD-iNSCs were determined (Fig. 1A). The expression levels of NSC markers were also confirmed by immunostaining of SOX2 and NESTIN (Fig. 1B). We examined the cumulative population doubling level of WT- and AD-iNSCs through continuous passaging from day 3 to day 18. Although there was no difference in marker expression between WT- and AD-iNSCs, the proliferation rate in AD-iNSCs were decreased compared to WT-iNSCs (Supplementary Fig. S1A, S1B). Cell proliferation was slightly decreased in AD-iNSCs compared to WT-iNSCs. In addition, to evaluate the multipotency of iNSCs, WT- and AD-iNSCs were differentiated for 7 days in neuronal media, and we confirmed neurons as evidenced by the expression of the neuron-specific class III beta-tubulin marker (TUJ1) (Fig. 1C). After that, to uncover the molecular cause of mitochondrial dysfunction in AD, we examined OXPHOS activity in neuronal mitochondrial function in AD patient cells. The levels of mitochondrial complex subunits III and V were decreased in AD-iNSCs-derived neurons compared to WT neurons (Fig. 1D, E). In agreement with dysfunctional ATP synthase, the level of ATP production was downregulated in AD-iNSCs, as shown by the reduced ATP production (Fig. 1F). Altered mitochondrial function can be suggested by ER-mitochondria apposition [28]. The location of dysregulated mitochondria was assessed by measuring the colocalization of ER and mitochondria. All mitochondria and the ER were tracked using MitoTracker and ER-Tracker (Fig. 1G). The colocalization of mitochondria and ER was increased in AD-iNSCs compared to WT-iNSCs (Fig. 1H). Thus, the altered localization of mitochondria and decreased OXPHOS level were indicative of mitochondria dysfunction in AD-iNSCs.

### APP-CTFs accumulation in AD patient-iNSCs altered mitochondrial function

**Fig. 2:**
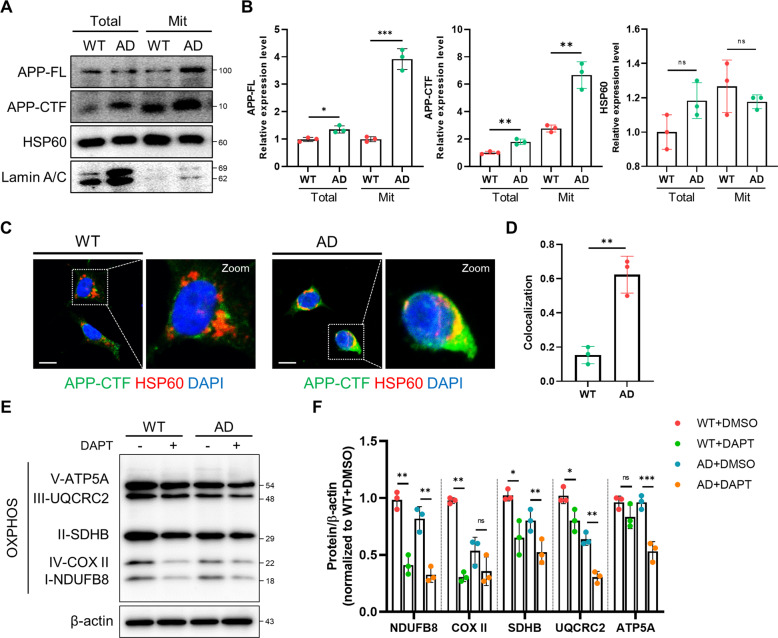
CTF accumulation is related to mitochondrial dysfunction. **A** Western blotting of APP and the mitochondrial marker HSP60 in whole-cell extracts and mitochondrial fractions. Full-length APP and CTFs detected with APP-CTF antibody. HSP60 and Lamin A/C antibodies were used as loading controls for whole-cell extraction and mitochondrial fractions, respectively. **B** Quantification of APP, APP-CTFs, and HSP60 in total extracts and mitochondrial fractions. **C** Immunostaining of WT- and AD-iNSCs using APP-CTF and HSP60 antibodies. Nuclei were stained with DAPI. Scale bars, 10 µm. **D** Quantification of the colocalization area using ImageJ in WT- and AD-iNSCs. **E** Western blotting of OXPHOS in vehicle- or γ-secretase inhibitor-treated WT- and AD-iNSCs using OXPHOS cocktail antibodies. **F** Quantification of the levels of NDUFB8, COX-II, SDHB, UQCRC2, and ATP5A in WT- and AD-iNSCs-treated vehicles (−) or γ-secretase inhibitor. Statistical analysis was performed by Student’s *t*-test. n.s not significant, *P < 0.05, **P < 0.01, and ***P < 0.001. The results are presented as the means ± SD.

Several studies have reported that APP-CTFs accumulation could represent an etiological trigger of AD pathology [9, 23, 29]. In particular, it has been reported that APP-CTFs could be responsible for mitochondrial alterations and dysfunction [21, 22]. First, we confirmed the level of APP-CTFs accumulation in whole lysates as well as the mitochondrial fraction (Fig. 2A). The level of CTFs in AD was upregulated in both the total lysates and mitochondrial fractions (Fig. 2B). We also demonstrated that the staining of APP-CTFs colocalized with the mitochondrial protein HSP60 in AD-iNSCs compared to WT-iNSCs (Fig. 2C, D). After that, to examine whether APP-CTFs are involved in mitochondrial dysfunction, we confirmed the level of oxidative phosphorylation after γ-secretase inhibition with DAPT treatment. All OXPHOS complex levels were downregulated after APP-CTFs accumulation induced by the γ-secretase inhibitor (Fig. 2E, F). These data demonstrate that APP-CTFs accumulation in mitochondria dysregulates mitochondrial function.

### Mitophagy failure in AD patient-iNSCs is related to APP-CTFs accumulation

**Fig. 3:**
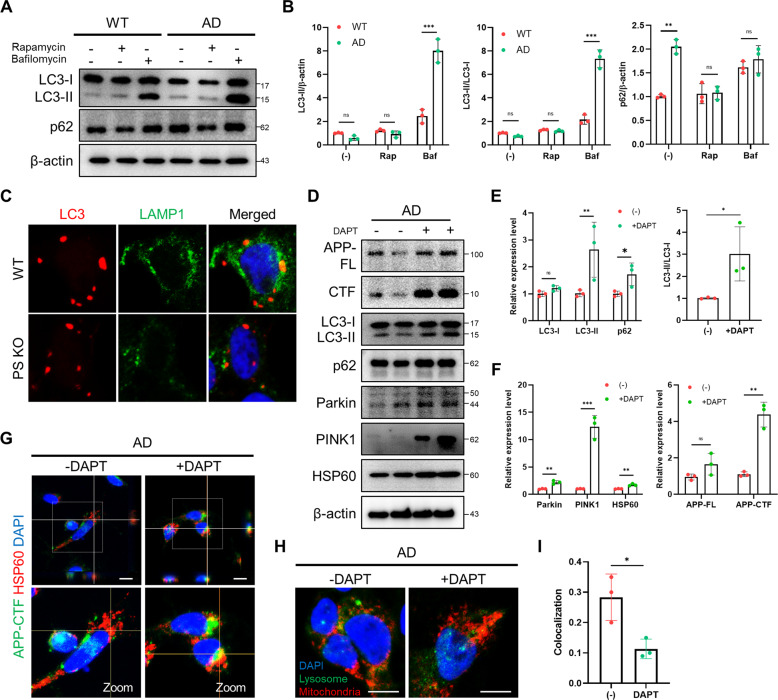
APP-CTFs accumulation triggers mitophagy dysfunction in AD-iNSCs. **A** Representative western blotting showing LC3 and p62 expression in WT- and AD-iNSCs treated or not with bafilomycin or rapamycin for 6 h. **B** Quantification of LC3-II, LC3-II/LC3-I, and the p62 ratio in WT- and AD-iNSCs with respect to control iNSCs under vehicle-treated conditions. **C** Representative images showing LC3 and LAMP1 in WT- and AD-iNSCs under basal conditions. **D** Representative western blotting showing APP, APP-CTFs, LC3, p62, Parkin, PINK1, HSP60, and β-actin expression in AD-iNSCs treated or not with DAPT for 24 h. **E** Quantification of autophagy-related markers, including LC3 and p62, in the presence of DAPT relative to vehicle treatment. **F** Quantification of APP-CTFs and mitophagy-related markers, including Parkin, PINK1, and HSP60, in the presence of 10 µM DAPT relative to vehicle treatment. **G** Representative z-stack images showing APP-CTF in green and HSP60 as a mitochondrial membrane marker in red in AD-iNSCs treated with 10 µM DAPT for 24 h. Scale bars, 20 µm. **H** Representative images showing LAMP1 (Lysosome marker) and MitoTracker (Mitochondria marker) in AD-iNSCs treated or not with DAPT for 24 h. Scale bars, 10 µm. **I** Quantification of colocalization area with LAMP1 and MitoTracker. Statistical analysis was performed by Student’s *t*-test. n.s not significant, *P < 0.05, **P < 0.01 and ***P < 0.001. The results are presented as the means ± SD.

Dysregulation of autophagy is associated with AD and involves the accumulation of misfolded proteins and damaged organelles, including mitochondria [30]. Perturbation in autophagy is measured by the synthesis and degradation of autophagosomes. While the elevation in the levels of p62 in cultured AD-iNSCs may be indicative of autophagy failure, it was necessary to examine this possibility using an autophagic flux assay [31]. We examined autophagic flux using bafilomycin A_1_ (Baf A_1_), which inhibits lysosomal acidification and degradation and increases LC3-II accumulation. We also assessed whether autophagy induction can be restored in AD-iNSCs after treatment with the autophagy inducer, mTOR-dependent rapamycin. In the absence conditions, we observed that the steady-state levels of LC3-I were slightly decreased, while p62 was elevated in AD-iNSCs compared to WT-iNSCs (Fig. 3A, B). The levels of LC3-II and p62 in AD-iNSCs were increased compared to those in WT-iNSCs after the presence of Baf A_1_ for 6 h. These results indicate that LC3-II and p62 accumulation is caused by the impaired degradation of autophagosomes. To assess whether LC3 is positively associated with the autolysosome structure, we examined the staining of LC3 and LAMP1 (Fig. 3C). The positive colocalization area of LC3 and LAMP1 was decreased in AD-iNSCs compared to WT-iNSCs. Next, to identify whether APP-CTFs accumulation in AD-iNSCs triggers mitophagy failure in AD-iNSCs, AD-iNSCs were treated with a chemical blockade of γ-secretase for 24 h (Fig. 3D). First, DAPT-treated cells showed increases in LC3-II, p62, and the LC3-II/LC3-I ratio compared to vehicle-treated cells, indicating a defective degradation according to APP-CTFs accumulation (Fig. 3E). AD-iNSCs treated with DAPT also showed defective mitophagy priming with significant increases in Parkin and PINK1 levels in AD-iNSCs (Fig. 3F). In addition, there was a significant increase in the mitochondrial protein HSP60 in DAPT-treated AD-iNSCs and an increase in APP-CTFs, demonstrating mitophagy failure according to APP-CTFs accumulation (Fig. 3F). We also demonstrated by immunofluorescence analysis whether APP-CTFs colocalize with the mitochondrial protein HSP60 in vehicle- and DAPT-treated AD-iNSCs (Fig. 3G). We confirmed that γ-secretase inhibition enhanced the colocalization of APP-CTFs and HSP60 in AD-iNSCs, indicating an accumulation of APP-CTFs in mitochondria. Additionally, we examined the accumulation of p62 and Ubiquitin in mitochondria as well as the recruitment of Parkin in mitochondria through immunofluorescence staining. The expression levels of p62 (Supplementary Fig. S2A), Parkin (Supplementary Fig. S2B), and Ubiquitin (Supplementary Fig. S2C) in mitochondria were increased in AD-iNSCs, showing damaged mitochondria that cannot be recycled due to a lack of degradation process by mitophagy. We also confirmed the reduced colocalization of mitochondria with lysosomes in DAPT-treated AD-iNSCs compared to vehicle-treated cells, suggesting that for defective degradation process in the lysosome (Fig. 3H, I). These results demonstrated that APP-CTFs were present in the mitochondrial compartment, indicating that APP-CTFs contribute to mitochondria dysfunction and defective degradation process of mitophagy.

### Generation of PSEN KO-iNSCs and characterization

**Fig. 4:**
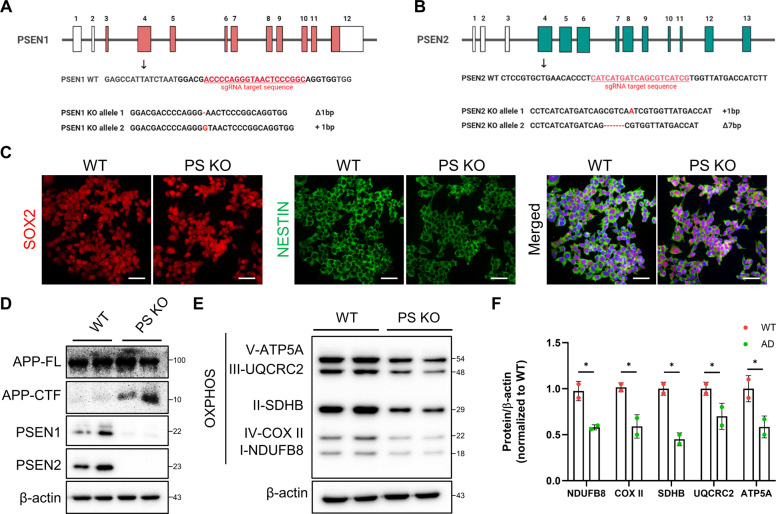
Deletion of PSEN in iNSCs and altered mitochondrial function in PSEN KO-iNSCs. **A** Schematic diagram showing the sgRNA target site for the generation of human PSEN1 knockout iNSCs by the CRISPR/Cas9 system. **B** Schematic diagram showing the sgRNA target site for the generation of human PSEN2 knockout iNSCs by the CRISPR/Cas9 system. **C** Representative images showing SOX2 and NESTIN in WT- and PSEN KO-iNSCs. Scale bars, 50 µm. **D** The levels of PSEN1, PSEN2, and APP-CTFs in iNSCs after PSEN deletion. **E** Western blotting of OXPHOS in vehicle- or γ-secretase inhibitor-treated WT- and PSEN KO-iNSCs using OXPHOS cocktail antibodies. **F** Quantification of the levels of NDUFB8, COX-II, SDHB, UQCRC2, and ATP5A in WT and PSEN KO-iNSCs. Statistical analysis was performed by Student’s *t*-test. *P < 0.05. The results are presented as the means ± SD.

Mutations in the *Presenilin* genes (*PSEN1 and PSEN2*) are associated with the major cause of familial Alzheimer’s disease [2]. APP is a major substrate of γ-secretase, which is critically involved in AD, and APP-CTFs are a direct substrate of γ-secretase that accumulates when γ-secretase activity is blocked. To further investigate whether mitochondrial-associated deficiency appears in the absence of PSEN in WT-iNSCs and to exacerbate the production of APP-CTFs from the cleavage of APP, we generated PSEN1/PSEN2 knockout iNSCs using CRISPR/Cas9 (Fig. 4A, B). Heterozygously targeted PSEN1 and PSEN2 KO iNSCs were confirmed by sequencing (Supplementary Fig. S3A, S3B). To assess the characterization of neural stem cells, we carried out immunofluorescence staining using antibodies against SOX2 and NESTIN in WT- and PSEN KO-iNSCs (Fig. 4C). Although the proliferation rate was decreased compared to WT-iNSCs, the cells could be maintained neural stem cell characterization (Supplementary Fig. S1C, S1D). We next performed western blotting to examine whether ablation of each exon leads to removal of protein level, PSEN1, and PSEN2, respectively. The protein level of both PSEN1 and PSEN2 was rarely expressed in PSEN KO-iNSCs, but APP-CTFs were expressed resulting in the blockade of γ-secretase activity (Fig. 4D).

### APP-CTFs accumulation in PSEN KO-iNSCs altered mitochondrial function

We next performed western blotting of OXPHOS to demonstrate whether the absence of PSEN can induce mitochondrial dysfunction in PSEN KO-iNSCs. The findings demonstrated that PSEN is involved in mitochondrial OXPHOS, in agreement with the downregulation of mitochondrial complex subunits. The levels of all mitochondrial complex subunits (I, II, III, IV, and IV) were decreased compared to those in WT-iNSCs (Fig. 4E, F). The downregulated result in PSEN KO-iNSCs was similar when γ-secretase activity was blocked with pharmacological inhibitors in WT- and AD-iNSCs.

### APP-CTFs accumulation is associated with mitophagy failure in PSEN KO-iNSCs

**Fig. 5:**
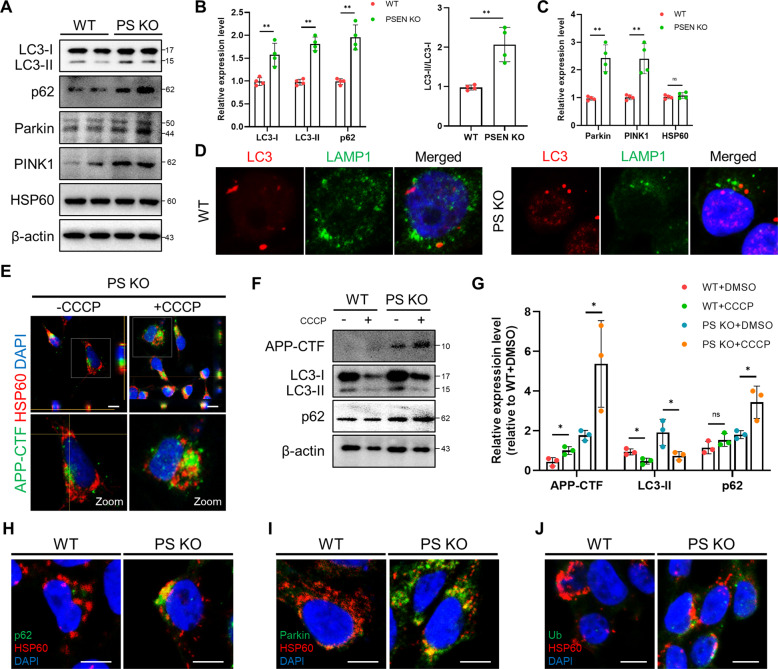
APP-CTFs accumulation triggers mitophagy failure in PSEN KO-iNSCs. **A** Representative western blotting showed mitophagy-related proteins, including LC3, p62, Parkin, PINK1, and HSP60, in WT- and PSEN KO-iNSCs. **B** Protein levels of LC3 and p62 in WT- and PSEN KO-iNSCs. **C** Protein levels of Parkin, PINK1, and HSP60 in WT- and PSEN KO-iNSCs. **D** Representative images showing LC3 and LAMP1 in WT- and PSEN KO-iNSCs under basal conditions. **E** Representative images of APP-CTFs and HSP60 protein in the absence or presence of CCCP (20 μM). Scale bars, 10 µm. **F** Representative western blot of LC3 and p62 in vehicle- or CCCP (20 μM)-treated WT- and PSEN KO-iNSCs. **G** Quantification of the levels in the absence or presence of CCCP. **H** Representative images of WT- and PSEN KO-iNSCs stained with anti-p62 (green) and anti-HSP60 (red). **I** Representative images of WT- and PSEN KO-iNSCs stained with anti-Parkin (green) and anti-HSP60 (red). **J** Representative images of WT- and PSEN KO-iNSCs stained with anti-Ub (green) and anti-HSP60 (red). Scale bar, 10 µm. Statistical analysis was performed by Student’s *t*-test. n.s not significant, *P < 0.05 and **P < 0.01. The results are presented as the means ± SD.

Damaged mitochondria are usually degraded through selective elimination by a specific autophagy process known as mitophagy. We also investigated the contribution of APP-CTFs accumulation to mitophagy in PSEN KO-iNSCs with APP-CTFs. The basal levels of LC3 and p62 were notably elevated in PSEN KO-iNSCs compared to WT-iNSCs (Fig. 5A, B). Our data showed a decrease in LC3-I but significant increases in LC3-II and the LC3-II/LC3-I ratio, indicating a defect in the clearance process of basal autophagy. In addition, the western blotting analysis showed significantly increased levels of Parkin and PINK1 in PSEN KO-iNSCs compared to WT-iNSCs (Fig. 5C). To examine degradation dysfunction, we addressed the colocalization of LC3 and LAMP1 in WT-iNSCs and PSEN KO-iNSCs (Fig. 5D). Reduced colocalization of LC3 with lysosomes was observed in PSEN KO-iNSCs compared to WT-iNSCs. Indeed, the accumulation of LC3-I, LC3-II and p62 together with Parkin and PINK1 in PSEN KO-iNSCs suggests that PSEN is critically involved in mitochondrial clearance. Next, we analyzed the amounts of APP-CTFs in the mitochondrial area after treatment with protonophore carbonyl cyanide m-chlorophenyl hydrazone (CCCP), which is the mitochondrial respiratory uncoupler that affects mitophagy (Fig. 5E). In western blotting analysis, both iNSCs were treated with CCCP, and the level of the degradation phase of autophagy was analyzed to assess whether the clearance process was altered in PSEN KO-iNSCs (Fig. 5F). The protein level of LC3-II was decreased in both WT and PSEN KO-iNSCs compared to vehicle-treated cells, whereas the level of p62 was increased in PSEN KO-iNSCs but not in WT-iNSCs (Fig. 5G). Additionally, we examined the expression of p62 and Ubiquitin in mitochondria as well as the recruitment of Parkin in mitochondria through immunofluorescence staining. We confirmed the slightly increased expression and colocalization of mitochondrial localization of p62 (Fig. 5H), Parkin (Fig. 5I), and Ubiquitin (Fig. 5J). Increased expression levels of p62 in PSEN KO-iNSCs reveal damaged mitochondria that cannot be recycled due to defects in the degradation process by mitophagy. These results indicate diminished mitophagy flux, where autophagosome synthesis was decreased and degradation was also decreased only in PSEN KO-iNSCs. Accordingly, PSEN has a crucial role in the control of mitophagy flux, and APP-CTFs may have a deleterious effect on this process.

### Neuronal differentiation was downregulated in PSEN KO-iNSCs

**Fig. 6:**
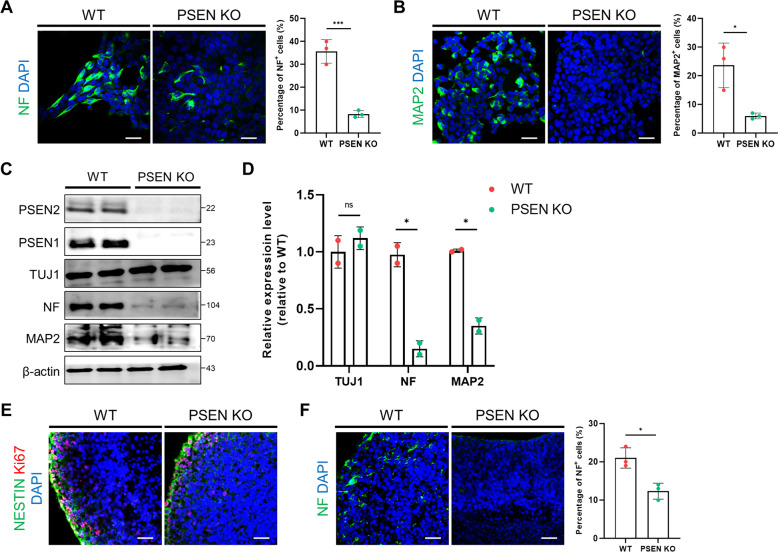
Neuronal differentiation was downregulated in 2D and 3D cultures of PSEN KO-iNSCs. **A** Representative images showing NF (neurofilament) in WT- and PSEN KO-iNSCs and quantification of the expression levels after neuronal differentiation for 7 days. Scale bars, 50 µm. **B** Representative immunofluorescence images showing MAP2 in WT- and PSEN KO-iNSCs and quantification of the expression levels after neuronal differentiation for 7 days. Scale bars, 50 µm. **C** Relative levels of proteins implicated in neuronal markers in WT- and PSEN KO-iNSCs after neuronal differentiation for 7 days. **D** Quantification of the levels of TUJ1, NF, and MAP2. **E** Representative immunofluorescence images showing the neural progenitor markers (NESTIN, Ki67) in WT and PSEN KO organoids at day 28. Scale bars, 50 µm. **F** Representative immunofluorescence images showing NF in WT and PSEN KO organoids at day 28. Scale bars, 50 µm. Quantification of the protein levels in PSEN KO organoids compared to WT organoids. Statistical analysis was performed by Student’s *t*-test. n.s not significant and *P < 0.05. The results are presented as the means ± SD.

It is known that PSEN plays a significant role in neuronal differentiation. Therefore, we examined the effect of neuronal differentiation in the absence of the PSEN gene using 2D and 3D modeling. In the 2D differentiation model, we induced iNSCs to differentiate into neurons followed by a differentiation protocol as described in the Methods. After 7 days of differentiation, the immunofluorescence levels of NF and MAP2 were drastically decreased in PSEN KO neurons compared to WT neurons (Fig. 6A, B). Western blot analysis revealed that PSEN KO neurons exhibited substantially decreased levels of mature neuronal markers (NF and MAP2) compared with WT neurons (Fig. 6C, D). However, there was no difference in the level of the immature neuron marker TUJ1. Finally, to estimate the neural level in the 3D brain organoid model, we followed the 3D culture protocol as described in the Methods section for 28 days [26]. Immunostaining analysis showed that the number of neural progenitor markers was similar in WT and PSEN KO organoids, but the neuronal level was markedly reduced in PSEN KO organoids (Fig. 6E, F). These results indicate that PSEN is an important factor in neuronal function as well as in the clearance process of cells.

## Discussion

These investigations of modeling FAD with patient-specific iNSCs revealed relatively early pathological manifestations of AD associated with APP metabolism. Our study focused on the effect of APP-CTFs in iNSCs derived from AD patient fibroblasts, and also investigated in PSEN KO-iNSCs. We demonstrated that mitochondrial dysfunction was upregulated in PSEN KO-iNSCs and AD patient-iNSCs compared to WT-iNSCs. Furthermore, APP-CTFs accumulation led to mitophagy defects, along with the failure of damaged mitochondrial clearance in both AD patient-iNSCs and PSEN KO-iNSCs. Accordingly, our data suggest that the measurement of APP-CTFs levels during APP metabolism could potentially be useful for the diagnosis of AD.

Our results regarding altered OXPHOS protein levels suggest mitochondrial dysfunction caused by molecular defects in OXPHOS. In previous reports, Aβ and p-Tau synergistically impaired mitochondrial function and energy homeostasis in vivo, resulting in the deregulation of complexes I and IV in OXPHOS [32, 33]. Aβ undermines several aspects of mitochondrial function, including electron transport chain (ETC), mitochondrial dynamics, ROS production, and mitochondrial transport [34]. The pathogenic forms of tau were also demonstrated to impair the mitochondrial respiratory chain complex, resulting in decreased ATP levels [35]. Previous results on altered protein and RNA expression of OXPHOS in AD patients reported that the OXPHOS pathway is one of the most significant factors and potential drivers of AD progression [36]. Using western blotting, we determined the differential expression of the mitochondrial genes; NADH dehydrogenase (complex I), succinate ubiquinone oxidoreductase (complex II), ubiquinol cytochrome c oxidoreductase (complex III), cytochrome oxidase subunit 1 (complex IV), and ATPase synthase (complex V), in AD-iNSCs. In addition, decreased OXPHOS levels were significantly downregulated in AD patient-iNSCs exposed to the γ-secretase inhibitor DAPT and PSEN KO-iNSCs, resulting in increased APP-CTFs expression. Our result of decreased ATP production was demonstrated concomitant with the deregulation of OXPHOS levels. Decreased expression of OXPHOS complexes is related to deregulated mitochondrial ATP synthase activity because complexes I and IV are necessary for ATP production. OXPHOS levels were downregulated in PSEN KO-iNSCs, suggesting that PSEN is a critical factor for mitochondrial homeostasis.

Altered mitochondrial-related factors cause defective mitophagy and autophagy in AD [37]. Many studies have focused on defective mitophagy linked to Aβ and p-Tau [18]. In this study, we demonstrated that APP-CTFs are closely related to mitophagy dysfunction, resulting in the failure to eliminate damaged mitochondria. Several studies have reported that CTFs of APP were neurotoxic, and transgenic mice expressing C99 or stereotaxically injected with APP-CTFs into the brain showed cortical atrophy, neurodegeneration, impairment in learning, and memory [7, 21, 22, 29]. APP-CTFs accumulation was also detected in brain samples from mild cognitive AD patients and has been suggested as a potential biomarker for AD [9]. Defective mitophagy phenotypes were examined in AD patients, and the PINK1-Parkin pathway was abrogated in PSEN KO-iNSCs, resulting in defective recycling of damaged mitochondria. Furthermore, marked increases in Parkin and PINK1 in mitochondria together with p62 and LC3-II in AD patient-iNSCs treated with γ-secretase inhibitor resulted in APP-CTFs accumulation. These results also suggest the risk of γ-secretase inhibitors as a therapeutic strategy for AD. This implies that targeting APP-CTFs with mitochondrial function and mitophagy could be a beneficial approach for ameliorating the development of pathologies as well as a new diagnostic biomarker in AD.

**Fig. 7:**
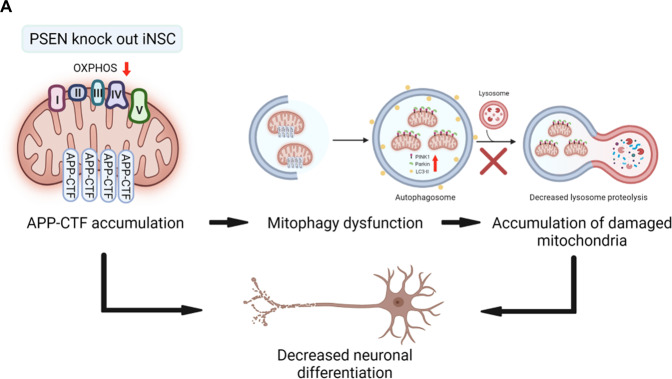
Summary figure. **A** APP-CTFs are involved in the AD-related pathologies including mitochondria dysfunction, mitophagy failure, and neuronal differentiation.

In summary, our findings indicate that iNSCs from FAD1 patients harboring PSEN1 mutations, and PSEN KO iNSC exhibit marked mitophagy failure as a result of exacerbated APP-CTFs accumulation in the neuronal model (Fig. 7A). Thus, these iNSC models could be a valuable tool to study AD pathology in further studies and therapeutic findings.

## Materials and methods

### iNSCs culture

Human iNSCs were used as reprogrammed cell lines, as described in our previous study. The commercial cell line hDFs was obtained from Life Technologies. These cells were cultured on PLO/FN-coated dishes and maintained in NSC maintenance media containing a 1:1 mixture of ReNcell media (Millipore, USA) and KnockOut DMEM/F12 supplemented with StemPro NSC SFM Supplement (Gibco/Life Technologies, USA) with (Sigma–Aldrich, Saint Louis, Missouri) and EGF (Sigma).

### 2D neuron differentiation

About 5000 cells of iNSCs per well were seeded onto PLO/FN-coated coverslips in 24-well plates, and the NSC maintenance medium was exchanged for random differentiation. After 3 days of random differentiation, the neuron differentiation media was added and sustained for 7 days. The neuron differentiation medium contained a 1:1 mixture of neurobasal medium (Gibco) and DMEM/F12 medium supplemented with B27 (Gibco), GMAX, brain-derived neurotrophic factor (BDNF; PeproTech, USA), glial cell line-derived neurotrophic factor (GDNF; PeproTech), forskolin (Sigma) retinoic acid (RA) (Sigma) and ascorbic acid (Sigma).

### Generation of AD fibroblast-derived iNSC

The use of human fibroblasts for cellular reprogramming was approved by the Institutional Review Board. On the day before transduction, AD fibroblasts (AG09035) (Coriell) were plated on 24-well tissue culture dishes. On the next day, the virus was added to the AD fibroblasts and spinfection was performed at 800 × *g* for 60 min at 20 °C. After transduction, the AD fibroblasts were incubated in DMEM/F12 (Life Technologies) containing 20% (v/v) fetal bovine serum (Life Technologies), and 1x primocin (InvivoGen, San Diego, CA, USA) for 3 days. On the third day, transduced cells were seeded onto Matrigel (Corning, NY, USA) coated six-well plates (Life Technologies). After cell attachment, the medium was changed with NSC medium (StemPro NSC medium with 1x supplement, 1x primocin, 20 ng/mL basic fibroblast growth factor (bFGF), and 20 ng/mL epidermal growth factor (EGF). On days 14–21, iNSC colonies were mechanically picked and cultured on a Matrigel-coated plate.

### Generation of PSEN double knockout iNSCs

Using the pCas-Guide CRISPR vector (OriGene, USA) as a backbone, a CRISPR vector for knocking out the PSEN1 and PSEN2 gene was cloned. Guide RNA sequences were ACCCCAGGGTAACTCCCGGC (PSEN1) and CATCATGATCAGCGTCATCG (PSEN2). In order to produce PSEN double knockout iNSCs, PSEN2 knockout iNSCs were produced and PSEN1 was additionally knockout sequentially. For gene knockout, CRISPR/Cas9 plasmid vectors for PSEN knockout were electroporated in iNSCs using the Neon transfection system (Life Technologies). After the electroporation, iNSC was cultured for 1–2 weeks to form a colony, and sequencing was performed for each colony. Sequencing was carried out in the order of genomic DNA preparation, PCR, sequencing, and analysis.

### Western blotting

The protein samples were prepared with the protein lysis buffer Pro-pro (Intron Biotechnology, Korea), and the mitochondrial fraction was isolated using a mitochondria isolation kit (Thermo Scientific, USA) according to the manufacturer’s specifications. The protein concentration was determined using bovine serum albumin (BSA) as the standard (Bio-Rad Laboratories, Hercules, USA). The proteins were separated via 10 or 15% sodium dodecyl sulfate-polyacrylamide gel electrophoresis (SDS-PAGE) and transferred to nitrocellulose membranes. After blocking with 3% bovine serum albumin in Tris-buffered saline with Tween (TBST: 20 mM Tris-HCl [pH 7.6], 137 mM NaCl, 1% Tween 20), the blots were probed overnight at 4 °C with the primary antibodies. The secondary antibodies, horseradish peroxidase (HRP)-conjugated antibodies (1:2000; Invitrogen, USA), were incubated with the membranes at room temperature for 1 h and detected using enhanced chemiluminescence (ECL) detection kit (GE Healthcare Life Science, UK). The primary antibodies used are listed in Supplementary Table 1.

### Quantitative reverse transcription-PCR (qRT–PCR)

Total RNA was extracted from cultured cells using TRIzol reagent (Invitrogen) following the manufacturer’s protocol. One microgram of RNA was reverse transcribed to first-strand cDNA using Superscript™ III reverse transcriptase (Invitrogen). For quantitative real-time PCR, relative mRNA levels were produced using SYBR-Green PCR Master Mix (Applied Biosystems, USA), and an ABI 7300 sequence detection system and the supplied software were used. The expression of each gene was normalized to that of the housekeeping gene, GAPDH. The primer sequences of each gene were described as follows: SOX2 (F: 5′-TGGCGAACCATCTCTGTGGT-3′ R: 5′-CCAACGGTGTCAACCTGCAT-3′), PAX6 (F: 5′-CCAGGGCAATCGGTGGTAG-3′ R: 5′-ATCGTTGGTACAGACCCCCT-3′), Nestin (F: 5′-CAGCGTTGGAACAGAGGTTGG-3′ R: 5′-TGGCACAGGTGTCTCAAGGGTAG-3′).

### Immunocytochemistry

For immunostaining, cells were fixed with 4% paraformaldehyde at room temperature for 15 min, permeabilized with 0.25% Triton X-100 in PBS for 10 min, and blocked with 5% normal goat serum for 1 h at room temperature. The cells were then stained with specific primary antibodies at 4 °C for overnight. Subsequently, Alexa 488- or 594-labeled secondary antibodies (Invitrogen) were stained at room temperature for 1 h, nuclei were stained with Hoechst 33258 for 5 min and images were produced using a confocal microscope (Nikon, Eclipse TE200).

### MitoTracker & ER-Tracker

All iNSCs were seeded at a density of 1 × 10^5^ cells per well in a 24-well PLO-FN-coated plate before the experiments with MitoTracker and ER-Tracker (Invitrogen). All steps followed the manufacturer’s protocol. Briefly, iNSCs were added to the stock solution of MitoTracker or ER-Tracker in 10 ml prewarmed media, giving final concentrations of 100 and 50 nM. After staining, cells were fixed with 3.7% formaldehyde at 37 °C for 10 min, permeabilized, and stained with DAPI solution for nuclear staining.

### ATP production assay

After cell lysis was deproteinized for sample preparation, ATP reaction mixtures and ATP measurements were performed using the ATP colorimetric/fluorescence assay kit (BioVision) as described in the manufacturer’s protocol. The ATP content was measured by running an internal standard. Absorbance was measured at OD 570 nm with a spectrophotometer in a microplate reader.

### Statistical analysis

The values of all data were expressed as the means ± SD. The statistical significance of differences was determined using GraphPad Prism version 9.0 (GraphPad Software, San Diego, CA, USA). For statistical analysis of experiments, two-tailed Student’s *t*-test or one-way ANOVA followed by Bonferroni’s test for multiple groups was used, as indicated in the figure legends. All samples were randomly selected and analyzed.

## Supplementary information

Supplementary information

Original data

Agreement about revised author list

## Data Availability

The datasets generated or analyzed during the current study are available from the corresponding author on reasonable request.

## References

[1] De Strooper B, Karran E (2016). The cellular phase of Alzheimer’s disease. Cell.

[2] Lanoiselee HM, Nicolas G, Wallon D, Rovelet-Lecrux A, Lacour M, Rousseau S (2017). APP, PSEN1, and PSEN2 mutations in early-onset Alzheimer disease: a genetic screening study of familial and sporadic cases. PLoS Med.

[3] Weggen S, Beher D (2012). Molecular consequences of amyloid precursor protein and presenilin mutations causing autosomal-dominant Alzheimer’s disease. Alzheimers Res Ther..

[4] Jiang Y, Sato Y, Im E, Berg M, Bordi M, Darji S (2019). Lysosomal dysfunction in Down Syndrome is APP-dependent and mediated by APP-betaCTF (C99). J. Neurosci..

[5] Kametani F, Haga S (2015). Accumulation of carboxy-terminal fragments of APP increases phosphodiesterase 8B. Neurobiol Aging.

[6] Kaur G, Pawlik M, Gandy SE, Ehrlich ME, Smiley JF, Levy E (2017). Lysosomal dysfunction in the brain of a mouse model with intraneuronal accumulation of carboxyl terminal fragments of the amyloid precursor protein. Mol Psychiatry.

[7] Berger-Sweeney J, McPhie DL, Arters JA, Greenan J, Oster-Granite ML, Neve RL (1999). Impairments in learning and memory accompanied by neurodegeneration in mice transgenic for the carboxyl-terminus of the amyloid precursor protein. Brain Res Mol Brain Res.

[8] Xu W, Weissmiller AM, White JA, Fang F, Wang X, Wu Y (2016). Amyloid precursor protein-mediated endocytic pathway disruption induces axonal dysfunction and neurodegeneration. J Clin Invest.

[9] Garcia-Ayllon MS, Lopez-Font I, Boix CP, Fortea J, Sanchez-Valle R, Lleo A (2017). C-terminal fragments of the amyloid precursor protein in cerebrospinal fluid as potential biomarkers for Alzheimer disease. Sci Rep..

[10] Swerdlow RH (2018). Mitochondria and mitochondrial cascades in Alzheimer’s disease. J Alzheimers Dis..

[11] Ohta S, Ohsawa I (2006). Dysfunction of mitochondria and oxidative stress in the pathogenesis of Alzheimer’s disease: on defects in the cytochrome c oxidase complex and aldehyde detoxification. J Alzheimers Dis..

[12] Ye X, Sun X, Starovoytov V, Cai Q (2015). Parkin-mediated mitophagy in mutant hAPP neurons and Alzheimer’s disease patient brains. Hum Mol Genet.

[13] Pickles S, Vigie P, Youle RJ (2018). Mitophagy and quality control mechanisms in mitochondrial maintenance. Curr Biol..

[14] Martin-Maestro P, Gargini R, A AS, Garcia E, Anton LC, Noggle S (2017). Mitophagy failure in fibroblasts and iPSC-derived neurons of Alzheimer’s disease-associated presenilin 1 mutation. Front Mol Neurosci..

[15] Harper JW, Ordureau A, Heo JM (2018). Building and decoding ubiquitin chains for mitophagy. Nat Rev Mol Cell Biol..

[16] Shaid S, Brandts CH, Serve H, Dikic I (2013). Ubiquitination and selective autophagy. Cell Death Differ..

[17] Cummins N, Tweedie A, Zuryn S, Bertran-Gonzalez J, Gotz J (2019). Disease-associated tau impairs mitophagy by inhibiting Parkin translocation to mitochondria. EMBO J.

[18] Fang EF, Hou Y, Palikaras K, Adriaanse BA, Kerr JS, Yang B (2019). Mitophagy inhibits amyloid-beta and tau pathology and reverses cognitive deficits in models of Alzheimer’s disease. Nat. Neurosci..

[19] Hu Y, Li XC, Wang ZH, Luo Y, Zhang X, Liu XP (2016). Tau accumulation impairs mitophagy via increasing mitochondrial membrane potential and reducing mitochondrial Parkin. Oncotarget.

[20] Kerr JS, Adriaanse BA, Greig NH, Mattson MP, Cader MZ, Bohr VA (2017). Mitophagy and Alzheimer’s disease: cellular and molecular mechanisms. Trends Neurosci..

[21] Vaillant-Beuchot L, Mary A, Pardossi-Piquard R, Bourgeois A, Lauritzen I, Eysert F (2021). Accumulation of amyloid precursor protein C-terminal fragments triggers mitochondrial structure, function, and mitophagy defects in Alzheimer’s disease models and human brains. Acta Neuropathol..

[22] Lauritzen I, Pardossi-Piquard R, Bourgeois A, Pagnotta S, Biferi MG, Barkats M (2016). Intraneuronal aggregation of the beta-CTF fragment of APP (C99) induces Abeta-independent lysosomal-autophagic pathology. Acta Neuropathol..

[23] Lauritzen I, Pardossi-Piquard R, Bourgeois A, Becot A, Checler F (2019). Does intraneuronal accumulation of carboxyl-terminal fragments of the amyloid precursor protein trigger early neurotoxicity in Alzheimer’s disease?. Curr Alzheimer Res.

[24] Zhang X, Li Y, Xu H, Zhang YW (2014). The gamma-secretase complex: from structure to function. Front Cell Neurosci..

[25] Yu KR, Shin JH, Kim JJ, Koog MG, Lee JY, Choi SW (2015). Rapid and efficient direct conversion of human adult somatic cells into neural stem cells by HMGA2/let-7b. Cell Rep..

[26] Lee SE, Shin N, Kook MG, Kong D, Kim NG, Choi SW (2020). Human iNSC-derived brain organoid model of lysosomal storage disorder in Niemann-Pick disease type C. Cell Death Dis..

[27] Sung EA, Yu KR, Shin JH, Seo Y, Kim HS, Koog MG (2017). Generation of patient specific human neural stem cells from Niemann-Pick disease type C patient-derived fibroblasts. Oncotarget.

[28] Area-Gomez E, Del Carmen Lara Castillo M, Tambini MD, Guardia-Laguarta C, de Groof AJ, Madra M (2012). Upregulated function of mitochondria-associated ER membranes in Alzheimer disease. EMBO J..

[29] Lauritzen I, Becot A, Bourgeois A, Pardossi-Piquard R, Biferi MG, Barkats M (2019). Targeting gamma-secretase triggers the selective enrichment of oligomeric APP-CTFs in brain extracellular vesicles from Alzheimer cell and mouse models. Transl Neurodegener..

[30] Ghavami S, Shojaei S, Yeganeh B, Ande SR, Jangamreddy JR, Mehrpour M (2014). Autophagy and apoptosis dysfunction in neurodegenerative disorders. Prog Neurobiol..

[31] Loos B, du Toit A, Hofmeyr JH (2014). Defining and measuring autophagosome flux-concept and reality. Autophagy.

[32] Rhein V, Song X, Wiesner A, Ittner LM, Baysang G, Meier F (2009). Amyloid-beta and tau synergistically impair the oxidative phosphorylation system in triple transgenic Alzheimer’s disease mice. Proc Natl Acad Sci USA.

[33] Koopman WJ, Distelmaier F, Smeitink JA, Willems PH (2013). OXPHOS mutations and neurodegeneration. EMBO J..

[34] Misrani A, Tabassum S, Yang L (2021). Mitochondrial dysfunction and oxidative stress in Alzheimer’s disease. Front Aging Neurosci..

[35] Perez MJ, Jara C, Quintanilla RA (2018). Contribution of tau pathology to mitochondrial impairment in neurodegeneration. Front Neurosci..

[36] Wang W, Zhao F, Ma X, Perry G, Zhu X (2020). Mitochondria dysfunction in the pathogenesis of Alzheimer’s disease: recent advances. Mol Neurodegener.

[37] Tran M, Reddy PH (2020). Defective autophagy and mitophagy in aging and Alzheimer’s disease. Front Neurosci..

