# Supplementary material for: Accumulation of APP-CTF induces mitophagy dysfunction in the iNSCs model of Alzheimer’s disease
Source: Cell Death Discov. 2022 Jan 10;8:1. doi: 10.1038/s41420-021-00796-3 (PMC8748980; doi:10.1038/s41420-021-00796-3)
Supplement: Supplementary file 1 — Supplementary information [file 41420_2021_796_MOESM1_ESM.docx]

**Supplementary Information**

Supplementary Table 1. List of antibodies used in this study.

| **Name** | **Species** | **Dilution** | **Vendor** | **Catalog #** |
| --- | --- | --- | --- | --- |
| APP-CTF | mouse | 1:500 | biolegend | 802803 |
| APP | Rabbit | 1:500 | Cell signaling | #2452 |
| Parkin | mouse | 1:1000 | Merck Milipore | MAB5512 |
| PINK1 | Rabbit | 1:1000 | Novus | BC100-494 |
| HSP60 | Rabbit | 1:1000 | Cell signaling | #12165 |
| NESTIN | mouse | 1:1000 | Millipore | MAB5326 |
| SOX2 | Rabbit | 1:1000 | Merck | ab5603 |
| Ki67 | Rabbit | 1:500 | abcam | ab15580 |
| TUJ1 | mouse | 1:500 | Biolegend | 801202 |
| LC3 | Rabbit | 1:500 | Novus | NB100 |
| P62 | mouse | 1:500 | BD bioscience | 610832 |
| Ubiquitin | Rabbit | 1:200 | abcam | ab7780 |
| LAMP1 | Mouse | 1:500 | Santa Cruz | SC-20011 |
| Neurofilament | Mouse | 1:500 | Cell signaling | #2836 |
| MAP2 | Mouse | 1:500 | Merck | MAB3418 |
| β-actin | mouse | 1:1000 | Cell signaling | #4967 |
| PSEN1 | Rabbit | 1:1000 | Cell signaling | #5643 |
| PSEN2 | Rabbit | 1:1000 | Cell signaling | #9979 |
| OXPHOS | mouse | 1:1000 | abcam | ab110411 |


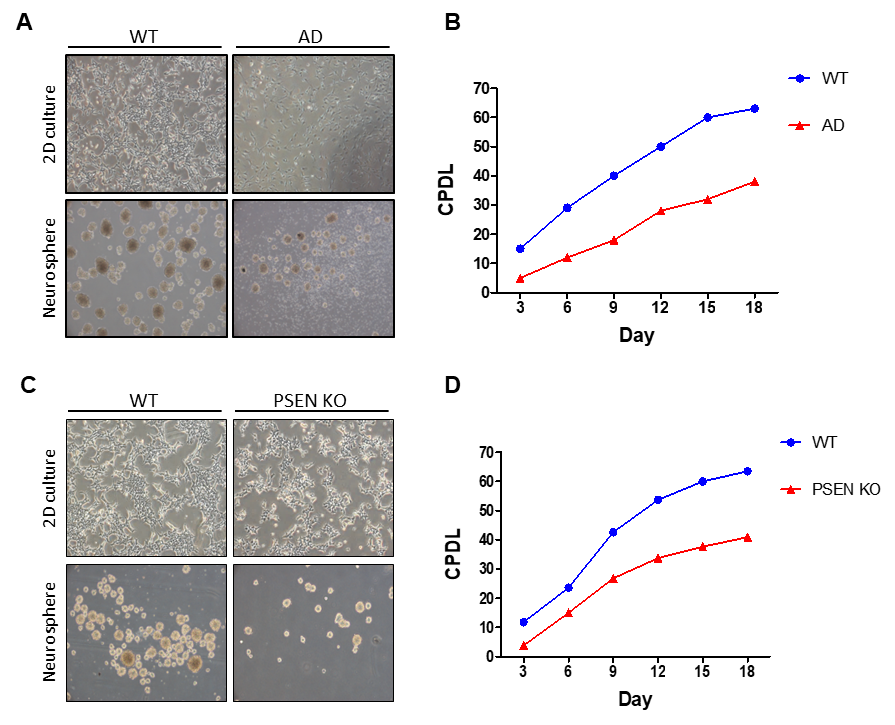


Supplementary figure S1. Proliferation rate of AD-iNSCs and PSEN KO-iNSCs.

(A) Morphology of WT- and AD-iNSCs in 2D culture and neurosphere formation. (B) Cumulative population doubling level (CPDL) of WT- and AD-iNSCs through continuous passaging from day 3 to day 18. (C) Morphology of WT- and PSEN KO-iNSCs in 2D culture and neurosphere formation. (D) Cumulative population doubling level (CPDL) of WT- and PSEN KO-iNSCs through continuous passaging from day 3 to day 18.


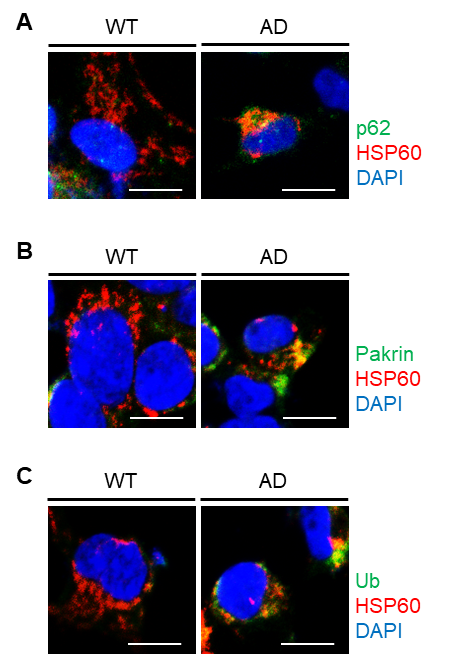


Supplementary figure S2. (A) Representative images of WT- and AD-iNSCs stained with anti-p62 (green) and anti-HSP60 (red). (B) Representative images of WT- and AD-iNSCs stained with anti-Parkin (green) and anti-HSP60 (red). (C) Representative images of WT- and AD-iNSCs stained with anti-Ub (green) and anti-HSP60 (red). Scale bars, 10 µm.


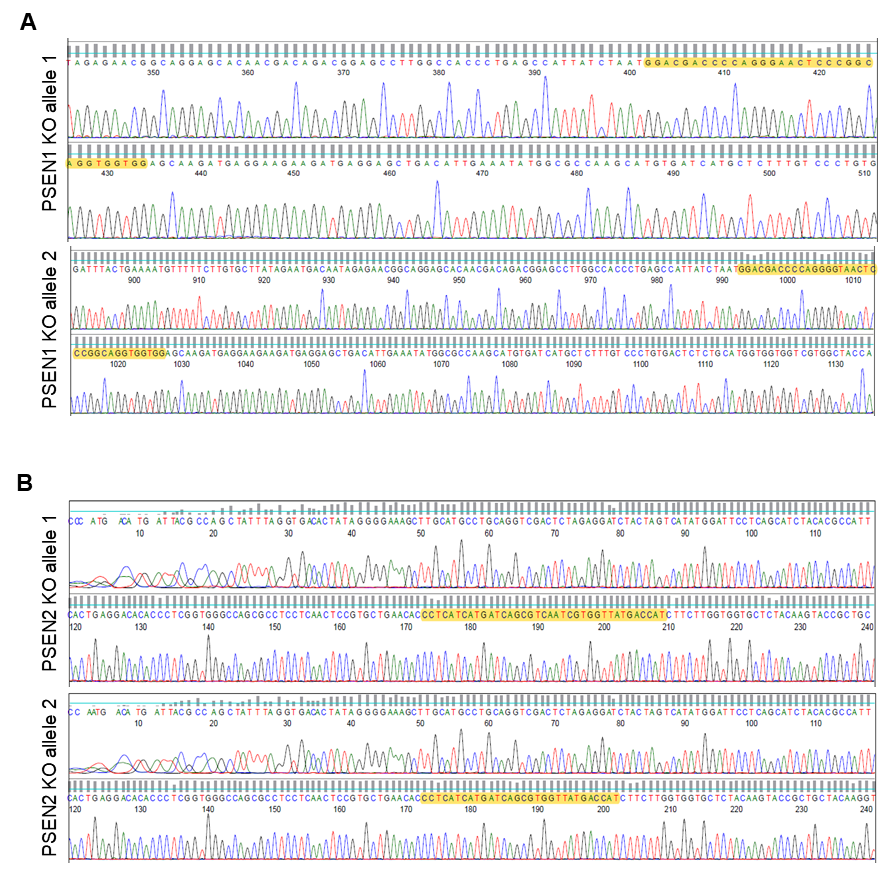


Supplementary figure S3. The genomic DNA sequence of PSEN1 and PSEN2 knockout iNSC.

(A) The genomic DNA sequence of PSEN1 knockout iNSCs. Targeting guide RNAs are shown in the yellow part of this panel. Sanger sequencing of PSEN1 knockout iNSC clones identified two deletions in exon 4 of PSEN1. Allele 1 is a 1-bp exonic deletion, and allele 2 is a 1-bp insertion.

(B) The genomic DNA sequence of PSEN2 knockout iNSCs. Targeting guide RNAs are shown in the yellow part of this panel. Sanger sequencing of PSEN2 knockout iNSC clones identified two deletions in exon 6 of PSEN2. Allele 1 is a 1-bp exonic insertion, and allele 2 is a 7-bp deletion.
