# Supplementary material for: Accumulation of APP-CTF induces mitophagy dysfunction in the iNSCs model of Alzheimer’s disease
Source: Cell Death Discov. 2022 Jan 10;8:1. doi: 10.1038/s41420-021-00796-3 (PMC8748980; doi:10.1038/s41420-021-00796-3)

Original data: Western blot

Figure 1

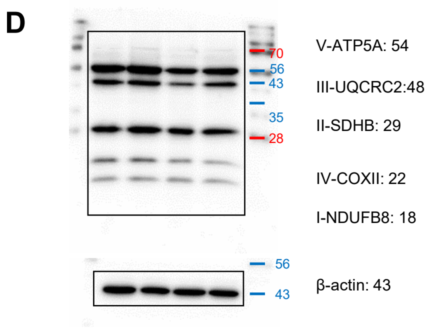

Figure 2

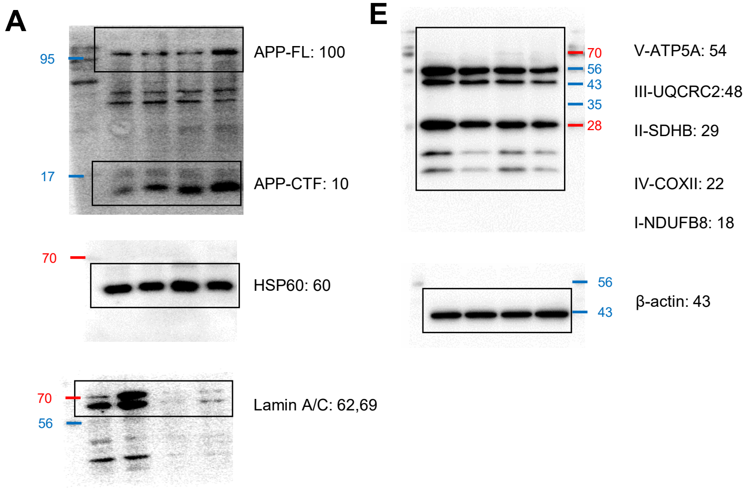

Figure 3

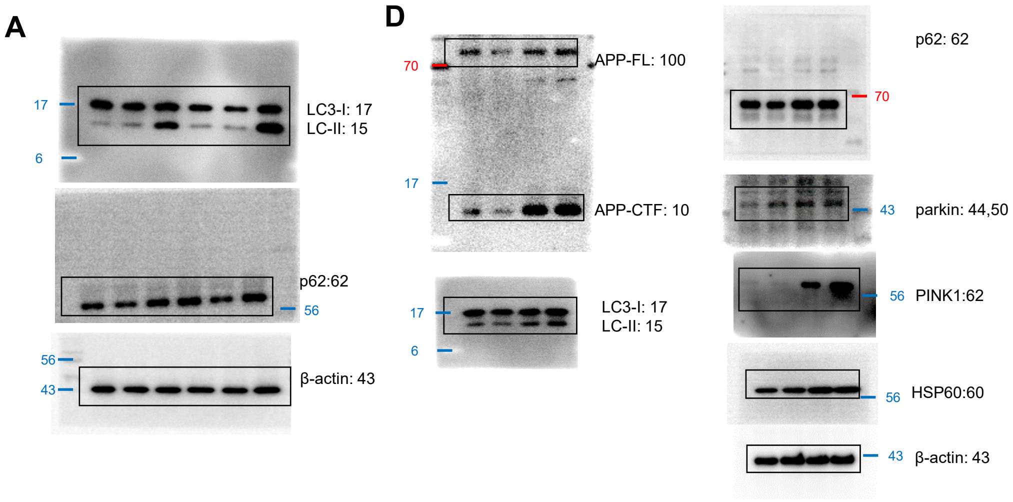

Figure 4

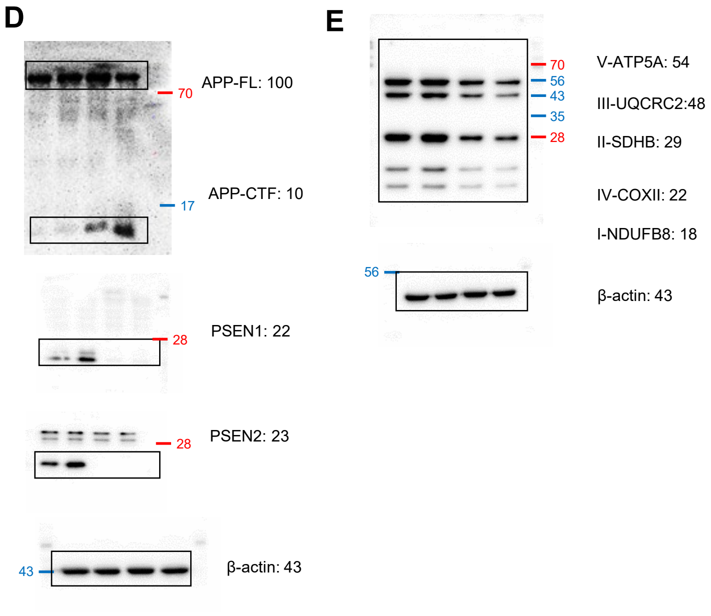

Figure 5

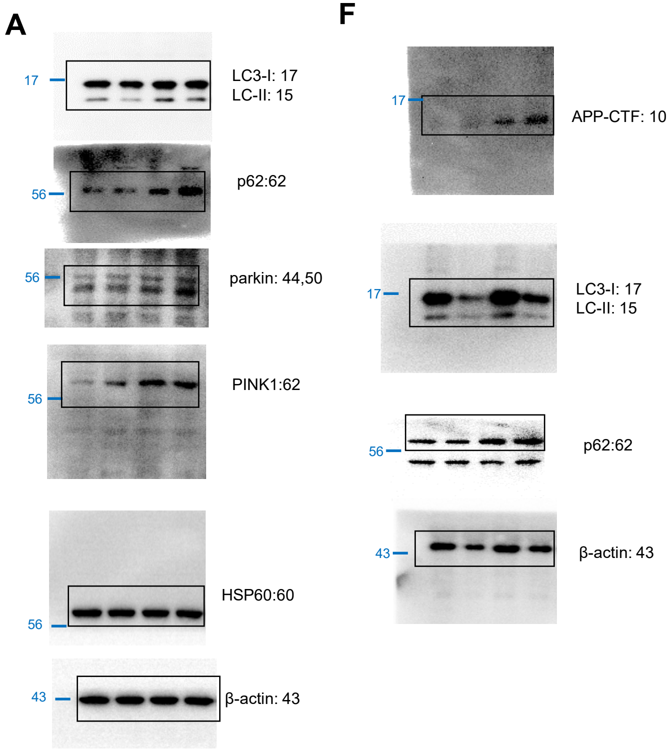

Figure 6

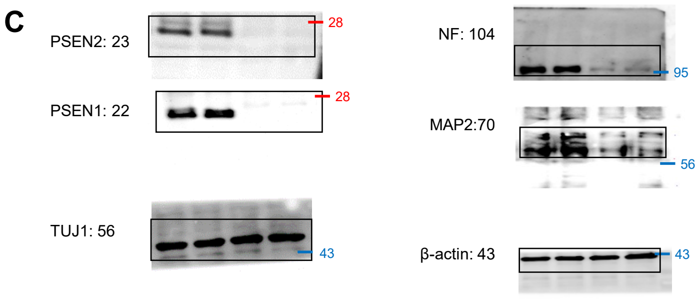

Original data: Immunostaining

Figure 1

B C G

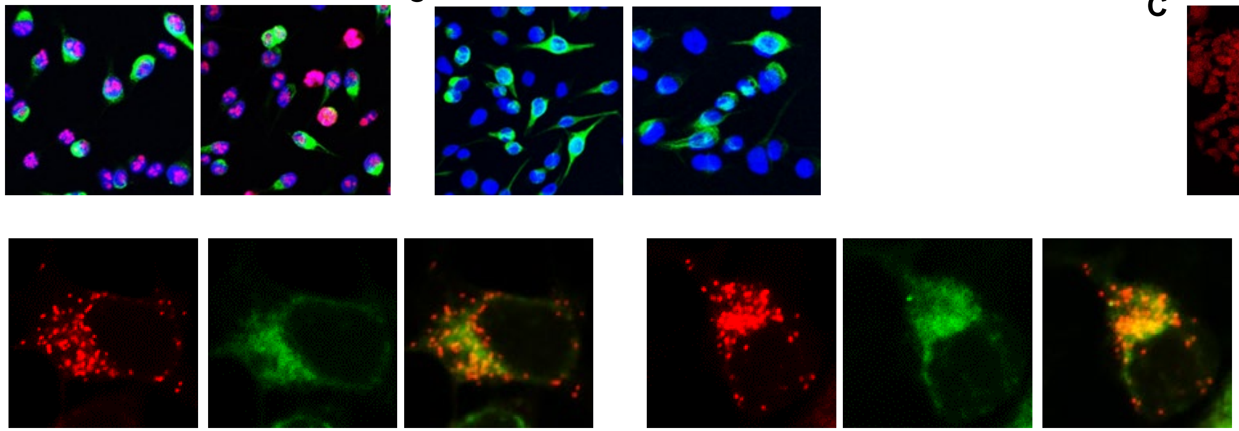

Figure 4

C

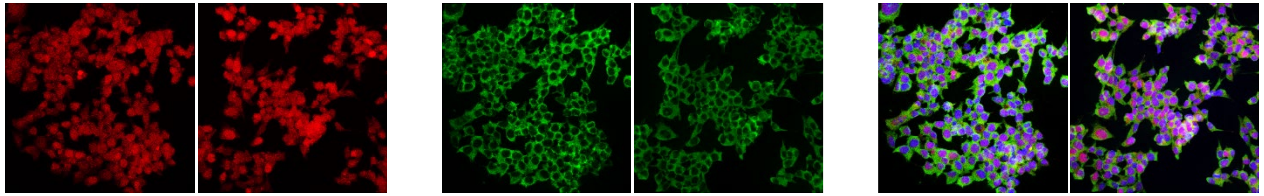

Figure 2

C

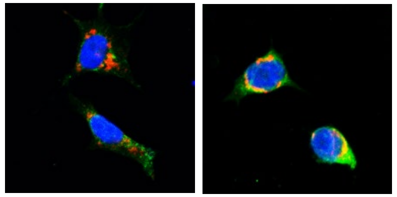

Figure 5

D E

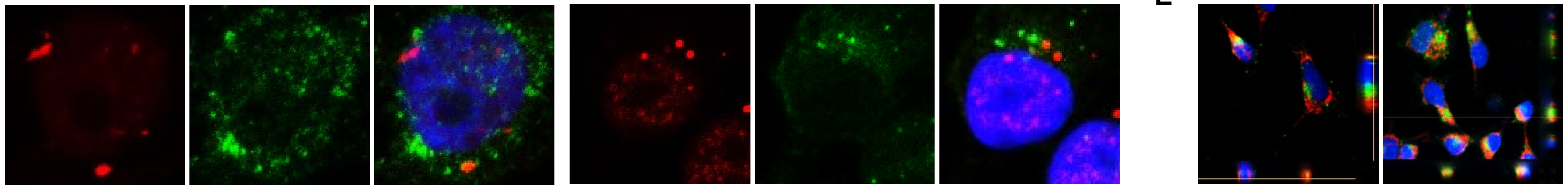

Figure 3

C G

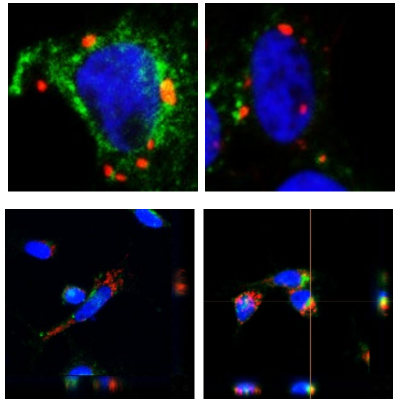

H

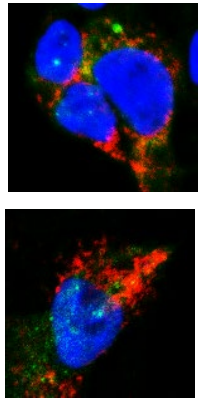

Figure 6

A B E F

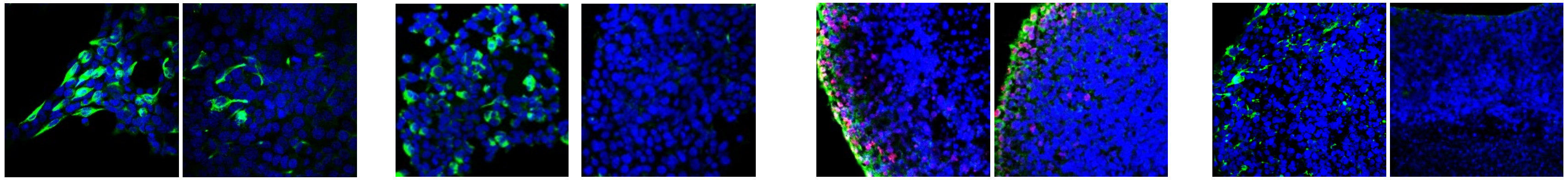

Supplement: Supplementary file 2 — Original data [file 41420_2021_796_MOESM2_ESM.pdf]
