# Supplementary material for: Accumulation of APP-CTF induces mitophagy dysfunction in the iNSCs model of Alzheimer’s disease
Source: Cell Death Discov. 2022 Jan 10;8:1. doi: 10.1038/s41420-021-00796-3 (PMC8748980; doi:10.1038/s41420-021-00796-3)
Supplement: Supplementary file 3 — Agreement about revised author list [file 41420_2021_796_MOESM3_ESM.pdf]

Request agreement about revised author list

9개의 메일

이승은 <eunlee05@snu.ac.kr> 2021년 12월 9일 오후 3:44
받는사람: 이승은 <eunlee05@snu.ac.kr>, 권대기 <dk.kwon@marurx.com>, sin2982@gmail.com, 공다솜 / 학생 / 수의학과 <dimsum0@snu.ac.kr>, 김남교 / 학생 / 수의학과 <ngkim93@snu.ac.kr>, 김희영 / 학생 / 수의학과 <khy728@snu.ac.kr>, 김민지 / 학생 / 수의학과 <minji9249@snu.ac.kr>, 최순원 / 연구원 / 수의과학연구소 <mszagm2007@snu.ac.kr>

Dear co-authors,

This is Seung-Eun Lee who is the first author of this study.
In our current study, "Accumulation of APP-CTF induces mitophagy dysfunction in the iNSCs model of Alzheimer's disease" CDDISCOVERY-21-2855R1, Soon Won Choi was added as an additional co-author. He contributed in data analysis during the review process.

In order for this to happen, we need a confirmation email from all of the co-authors listed in this manuscript.

Please reply to this email whether you confirm or deny the addition of Soon Won Choi as a co-author in this manuscript.

Thank you

Regards,

Seung Eun Lee

Adult Stem Cell Research Center
College of Veterinary Medicine, Seoul National University
#85-728, Seoul National University, 1 Gwanak-ro, Gwanak-gu, Seoul,

South Korea

C.P. : +82-10-2302-3386
Office: +82-2-880-1298
FAX : +82-2-876-7610

신나리 <sin2982@gmail.com> 2021년 12월 9일 오후 3:45
받는사람: 이승은 <eunlee05@snu.ac.kr>

Hi, Seung Eun.

I agree the addition of Soon Won Choi as a co-atuhor in this manuscript.

Thank you.

2021-12-09 오후 3:44에 이승은 이(가) 쓴 글:
[받은메일 숨김]

--
안녕하세요.
서울대학교 수의과대학 강경선 교수님 연구실의 신나리입니다.

김희영 / 학생 / 수의학과 <khy728@snu.ac.kr> 2021년 12월 9일 오후 3:49
받는사람: 이승은 <eunlee05@snu.ac.kr>

Hello,

I would confirm the addition of Soon Won Choi as a co-author in this manuscript.

Thanks & Regards

2021년 12월 9일 (목) 오후 3:44, 이승은 <eunlee05@snu.ac.kr>님이 작성:
[받은메일 숨김]

Soon Won Choi <mszagm2007@snu.ac.kr> 2021년 12월 9일 오후 3:49
받는사람: 이승은 <eunlee05@snu.ac.kr>

Dear Lee,

I agree with the addition of Soon Won Choi as a co-atuhor in this manuscript.

Best regards,

Soon Won Choi

2021년 12월 9일 (목) 오후 3:44, 이승은 <eunlee05@snu.ac.kr>님이 작성:

> Dear co-authors,
> [받은메일 숨김]
>
> Thank you
>
> Regards,
>
> [받은메일 숨김]

김민지 / 학생 / 수의학과 <minji9249@snu.ac.kr> 2021년 12월 9일 오후 3:50
받는사람: 이승은 <eunlee05@snu.ac.kr>

Hi

I confirm the addition of Soon Won Choi as a co-author in this manuscript.

2021년 12월 9일 (목) 오후 3:44, 이승은 <eunlee05@snu.ac.kr>님이 작성:

> Dear co-authors,
> [받은메일 숨김]
>
> Thank you
>
> Regards,
>
> [받은메일 숨김]

이승은 <eunlee05@snu.ac.kr> 2021년 12월 9일 오후 3:56
받는사람: 이승은 <eunlee05@snu.ac.kr>, 권대기 <dk.kwon@marurx.com>, sin2982@gmail.com, 공다솜 / 학생 / 수의학과 <dimsum0@snu.ac.kr>, 김남교 / 학생 / 수의학과 <ngkim93@snu.ac.kr>, 김희영 / 학생 / 수의학과 <khy728@snu.ac.kr>, 김민지 / 학생 / 수의학과 <minji9249@snu.ac.kr>, 최순원 / 연구원 / 수의과학연구소 <mszagm2007@snu.ac.kr>

I agree with the addition of Soon Won Choi as a co-atuhor in this manuscript.

Regards,
Seung-Eun Lee

2021년 12월 9일 (목) 오후 3:44, 이승은 <eunlee05@snu.ac.kr>님이 작성:

> Dear co-authors,
> [받은메일 숨김]
>
> Thank you
>
> Regards,

[받은메일 숨김]

--
[받은메일 숨김]

공다솜 <dimsum0@snu.ac.kr> 2021년 12월 9일 오후 3:58
받는사람: 이승은 <eunlee05@snu.ac.kr>

I agree with the addition of Soon Won Choi as a co-atuhor in this manuscript.

Dasom Kong

2021년 12월 9일 (목) 오후 3:44, 이승은 <eunlee05@snu.ac.kr>님이 작성:

> Dear co-authors,
> [받은메일 숨김]
>
> Thank you
>
> Regards,
>
> [받은메일 숨김]

--
안녕하세요, 서울대학교 강경선 교수님 연구실 공다솜입니다.

감사합니다.

Dasom Kong
Laboratory of public health
College of Veterinary Medicine, Seoul National University

E-mail : dimsum0@snu.ac.kr
Tel : +82-2-880-1298
+82-10-5030-9100

권대기 <dk.kwon@marurx.com> 2021년 12월 9일 오후 4:09
받는사람: 이승은 <eunlee05@snu.ac.kr>, 공다솜 / 학생 / 수의학과 <dimsum0@snu.ac.kr>, 김남교 / 학생 / 수의학과 <ngkim93@snu.ac.kr>, 김희영 / 학생 / 수의학과 <khy728@snu.ac.kr>, 김민지 / 학생 / 수의학과 <minji9249@snu.ac.kr>, 최순원 / 연구원 / 수의과학연구소 <mszagm2007@snu.ac.kr>, sin2982@gmail.com

I agree with the addition of Soon Won Choi as a co-atuhor in this manuscript.

Regards,

Daekee Kwon

권 대 기 이사/수의학박사 연구소 I 연구소장/CTO

Daekee, Kwon Director/Ph.D. Maru Therapeutics Research Institute (MTRI) I Research Director/CTO

T +82.2.6953.5459
F +82.2.6953.5389
M +82.10.3269.4810
E dk.kwon@marurx.com
A 05854 서울특별시 송파구 법원로 114 엠스테이트 B동 12층 1209호
B-1209, Mstate, 114, Beobwon-ro, Songpa-gu, Seoul 05854, Korea

MARU THERAPEUTICS

보내는사람 : 이승은 <eunlee05@snu.ac.kr>

받는사람 : 이승은 <eunlee05@snu.ac.kr>,권대기 <dk.kwon@marurx.com>,공다솜 / 학생 / 수의학과 <dimsum0@snu.ac.kr>,김남교 / 학생 / 수의학과 <ngkim93@snu.ac.kr>,김희영 / 학생 / 수의학과 <khy728@snu.ac.kr>,김민지 / 학생 / 수의학과 <minji9249@snu.ac.kr>, 최순원 / 연구원 / 수의과학연구소 <mszagm2007@snu.ac.kr>,sin2982@gmail.com

보낸 날짜 : 2021-12-09 15:44:10

제목 : Request agreement about revised author list
[받은메일 숨김]

김남교 <ngkim93@snu.ac.kr> 2021년 12월 9일 오후 4:12
받는사람: 이승은 <eunlee05@snu.ac.kr>

To Seung-Eun Lee

I agree with the addition of Soon Won Choi as a co-atuhor in this manuscript.

Thank you

Regards, Nam Gyo Kim

2021년 12월 9일 (목) 오후 3:44, 이승은 <eunlee05@snu.ac.kr>님이 작성:

> Dear co-authors,
> [받은메일 숨김]
>
> Thank you
>
> Regards,
>
> [받은메일 숨김]
